# Comparison between Brain Computed Tomography Scan and Transcranial Sonography to Evaluate Third Ventricle Width, Peri-Mesencephalic Cistern, and Sylvian Fissure in Traumatic Brain-Injured Patients

**DOI:** 10.3389/fneur.2017.00044

**Published:** 2017-02-15

**Authors:** Raphael A. G. Oliveira, Marcelo de Oliveira Lima, Wellingson Silva Paiva, Luiz Marcelo de Sá Malbouisson, Manuel Jacobsen Teixeira, Edson Bor-Seng-Shu

**Affiliations:** ^1^Intensive Care Unit, Discipline of General Surgery and Trauma, Hospital das Clínicas, São Paulo University Medical School, São Paulo, Brazil; ^2^Division of Neurosurgery, Hospital das Clínicas, São Paulo University Medical School, São Paulo, Brazil

**Keywords:** traumatic brain injury, transcranial color-coded sonography, midline shift, perimesencephalic cisterns, sylvian fissure

## Abstract

**Introduction:**

Transcranial color-coded duplex sonography (TCCS) may help guide multimodal monitoring in the neurocritical setting. It may provide indirect information about intracranial hypertension, such as midline shift, third ventricle width, and peri-mesencephalic cistern obliteration. We aim to assess the agreement between brain computed tomography scan (CT scan) and TCCS in traumatic brain injury (TBI) patients.

**Methods:**

In this retrospective cross-sectional observational study, TCCS was performed within 6 h before a brain CT scan. Only the first CT and TCCS after ICU admission were included. The agreement between the CT scan and TCCS was assessed by Bland–Altman plots and evaluating the intraclass correlation coefficient.

**Results:**

Overall, 15 consecutive patients were included (80% male, 42 ± 23 years of age, Glasgow Coma Score 5 [4,6]). The mean difference between the brain CT scan and TCCS in measuring the midline shift was 0.30 ± 2.1 mm (intraclass correlation coefficient: 0.93; *p* < 0.01). An excellent correlation was also observed between the methods in assessing the third ventricle width (intraclass correlation coefficient: 0.88; *p* < 0.01). Bland–Altman plots did not show any systematic bias in either agreement analysis. TCCS showed good accuracy in predicting non-compressed peri-mesencephalic cisterns (AUC: 0.83, 95% CI 0.46–1.0) and the presence of the Sylvian fissure (AUC: 0.91, 95% CI 0.73–1.0) on CT scan.

**Conclusion:**

TCCS is a promising tool and may be an alternative to CT scans for evaluating TBI patients.

## Introduction

Severe traumatic brain injury (TBI) remains an important cause of death and severe disability in adults. Thus, serial computed tomography (CT) brain scan imaging plays a crucial role in monitoring patients and helping guide intensive care management in the acute TBI phase (1). In addition to the identification of neurosurgical lesions, brain CT scanning provides important information about intracranial pressure features, such as midline structural shifts, peri-mesencephalic cisterns, and third ventricle widths (2).

In addition to brain CT scans, transcranial color-coded sonography (TCCS) has been commonly applied in neurocritical care scenarios as a valuable tool to monitor acute brain-injured patients because of its non-invasive feature and bedside application. TCCS has been widely used as a standard technique to evaluate the cerebral blood flow velocity of the intracranial arterial system, mainly in the acute stroke setting (3–6). However, TCCS also allows an accurate description of the cerebral anatomy, including hematomas or ventricular enlargements, in patients with intact skulls. Additionally, several authors demonstrated that midline structural displacement and hyperdense lesions could be accurately measured by TCCS compared to brain CT scanning in acute brain-injured patients, with either intact skulls or decompressive craniectomy (7).

However, it is unknown whether TCCS is able to assess features usually seen in brain CT scans to evaluate intracranial hypertension, such as compressed peri-mesencephalic cisterns, third ventricle obliteration, and Sylvian fissure effacement (8). Thus, considering the potential role of TCCS to evaluate intracranial hypertension in TBI, we hypothesized that this technique could be used as an adjuvant neuroimaging methodology in scenarios where the patient cannot be transported to the CT room or if CT is not available.

Thus, in this study, we aimed to evaluate the agreement between cerebral CT scanning and TCCS to visualize third ventricle widths, Sylvian fissure effacement, and peri-mesencephalic cistern compression in severe traumatic brain-injured patients.

## Materials and Methods

We performed a cross-sectional retrospective observational study at the 18-bed Surgical Emergencies and Trauma Intensive Care Unit of the General Hospital of the University of São Paulo Medical School. The data were collected between July 2015 and October 2015. All consecutive patients with severe TBI admitted to the ICU were evaluated during this period. The hypotheses were generated before data analysis and after data collection. The ethics committee approved the study (CAPPesq), and written informed consent was waived because of the study’s rigorous observational design.

**Figure 1:**
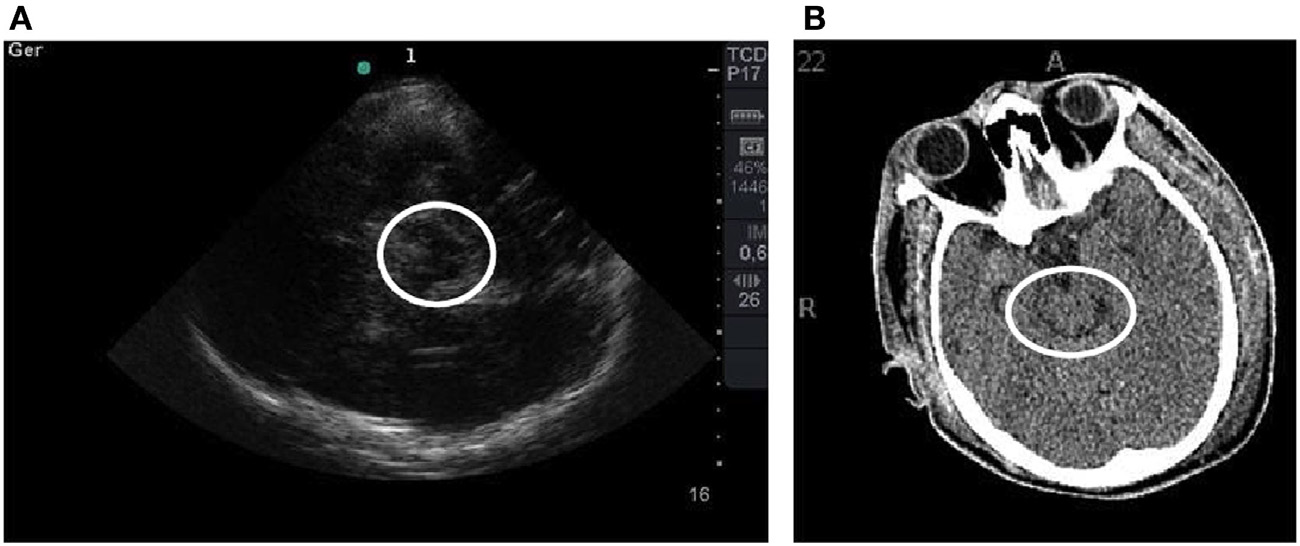
**(A)** TCCS image shows the mesencephalic plane with a “butterfly-wing” structure surrounded by a white circle. Note the hyperechogenic margins. **(B)** Corresponding tomographic imaging is shown.

**Figure 2:**
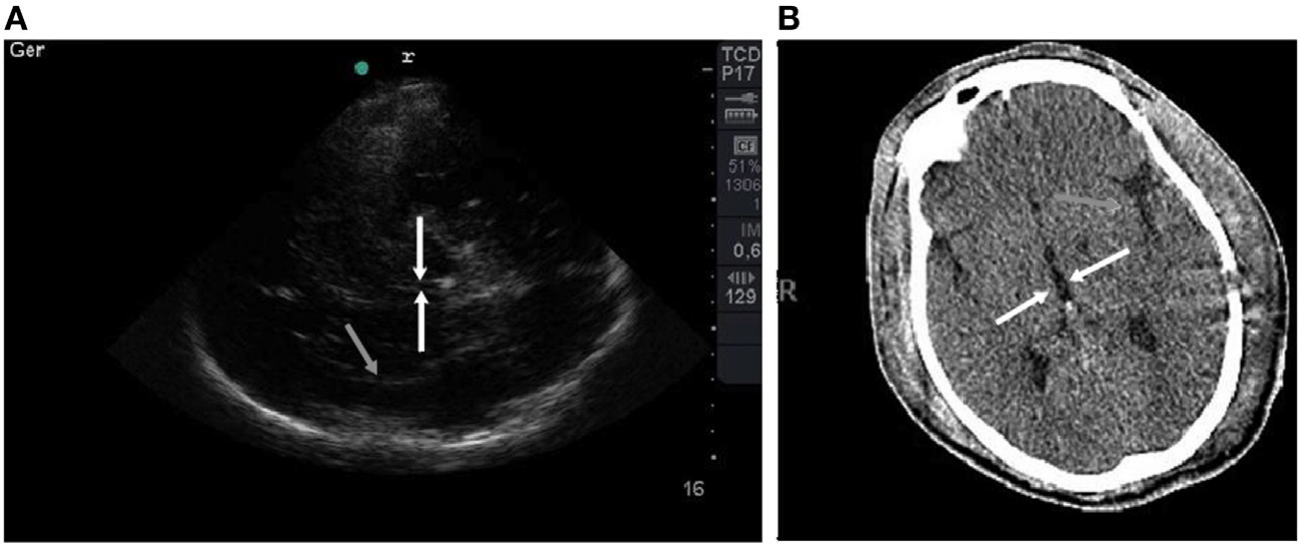
**(A)** TCCS image shows the diencephalic plane with third ventricle with hyperechogenic margins emphasized by white arrows. The Sylvian fissure is shown highlighted by a gray arrow. **(B)** Corresponding tomographic imaging is shown.

Only the first brain CT scan and TCCS of each patient was included. All exams were performed conforming to medical decisions and within 24 h after ICU admission. The Marshall classification was used to assess the severity of the injury (9). An experienced neuro-intensive care physician who was blinded to the TCCS results evaluated the CT scans. TCCS was performed within 6 h before the CT scan by an experienced single-operator using a Sonosite^®^ device with a 2.5 MHz phased array transducer probe with the transcranial Doppler setting through the transtemporal acoustic bone window (axial plane), as described by Seidel (10). Initially, the mesencephalon was tracked with a classic “butterfly-wing” structure (mesencephalic plane). We supposed that well-defined mesencephalon margins on TCCS denoted the peri-mesencephalic cisterns clearly seen on CT scan, which would indicate a non-compressed state (Figure 1). Later, the probe was moved 10° upward to locate the third ventricle with its hyperechogenic margins and the surrounding hypoechogenic talami and posterior hyperechogenic pineal gland (diencephalic plane). We also observed the Sylvian fissure on this plane and characterized it as the hyperechogenic structure perpendicular to the probe on the contralateral side, nearly to the skull (Figure 2). The third ventricle width was measured in the axial plane between its hyperechogenic margins. We presumed that in cases of high cerebral complacency, we would observe the presence of the Sylvian fissure on TCCS, as is normally seen on brain CT scans in this scenario. Furthermore, the presence of the third ventricle on TCCS was interpreted as indicating a normal cerebral complacency status.

The distance between the probe and the center of the third ventricle was measured along a line perpendicular to the walls of the third ventricle from both the ipsilateral and contralateral sides, called the *A* and *B* distances, respectively. The midline shift was calculated by (*A*−*B*)/2 (11).

Clinical data such as age, gender, Simplified Acute Physiology Score 3 (SAPS 3), Glasgow Coma Score, TCCS results, and hospital mortality were extracted from our electronic database to perform a baseline analysis.

### Statistical Analysis

Parametric variables were expressed as the mean (SD), and non-parametric variables were expressed as the median [IQ]. Inferential analysis was performed to evaluate the agreement between the CT scan and TCCS in measuring the midline shift and third ventricle width. The agreement between the techniques was evaluated by analyzing the intraclass correlation coefficient (>0.75 was considered a good correlation) and Bland–Altman plots (12). The area under the curve, specificity, and sensitivity were analyzed to evaluate the performance of TCCS in predicting non-compressed peri-mesencephalic cisterns and the presence of the Sylvian fissure compared to the CT scan. The correlation between parametric variables was evaluated using Pearson’s correlation. *p* < 0.05 was considered statistically significant. Statistical analyses were conducted using SPSS 19 (SPSS Inc., Chicago, IL, USA) and the R Project.

## Results

Fifteen consecutive severe traumatic brain-injured patients with intact skulls were evaluated. The mean age was 42 ± 23 years, and 80% were male. The SAPS 3 score was 56 ± 12. The Glasgow coma score at the trauma scene after initial stabilization was 5 [4,6]. The observed hospital mortality rate was 53%. The individual characteristics at baseline are presented in Table 1.

**Table 1:** **Individual characteristics at baseline and outcome**.

Patient	Age, years	Gender	Simplified Acute Physiology Score 3	GCS^a^	Marshall CT	Hospital mortality
1	27	F	63	7	III	Yes
2	22	M	48	4	III	Yes
3	15	M	53	5	V	Yes
4	32	F	51	5	V	Yes
5	79	M	67	5	V	Yes
6	23	M	82	3	III	No
7	90	F	65	5	V	No
8	42	M	54	8	II	No
9	52	M	41	3	V	No
10	20	M	56	4	V	No
11	51	M	68	6	VI	Yes
12	38	M	40	5	II	No
13	67	M	66	8	V	No
14	61	M	67	4	V	Yes
15	22	M	42	6	III	Yes

*^a^Glasgow coma score at hospital admission*.

**Figure 3:**
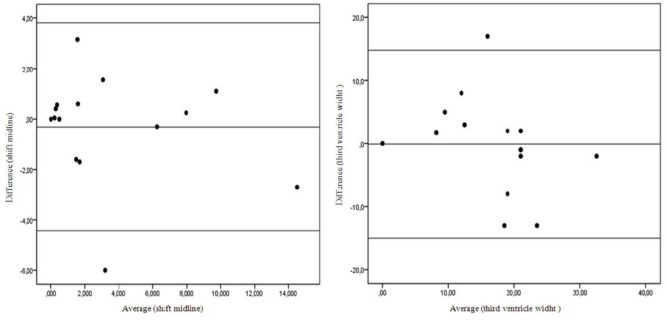
**Bland–Altman plot shows measurement agreement between TCCS and brain computed tomography scan (CT scan)**.

An excellent correlation was observed between CT scanning and TCCS concerning midline structural shifts (*b*: 0.978, *p* < 0.01) and third ventricle widths (*b*: 0.85, *p* = 0.01). The mean difference between the CT scan and TCCS was −0.308 [95% confidence interval (CI), −4.42–3.80, *p* = 0.57] and −0.08 (95% CI, −14.97–14.81, *p* = 0.96) for the measure of midline structure shift and third ventricle width, respectively. The agreement between the methods for both measures was excellent (Table 2), and no systematic bias was observed on the Bland–Altman plot (Figure 3).

**Table 2:** **Results of comparison between TCCS and brain computed tomography scan (CT scan)**.

Variable	TCCS^a^	Computed tomography	Intraclass correlation (95% CI)	*p*
Shift medium line, mm	3.6 ± 4.5	3.3 ± 4.17	0.93 (0.81, 0.98)	<0.01
Third ventricle width, mm	35.5 ± 12	33.1 ± 14	0.88 (0.63, 0.96)	<0.01

*^a^Transcranial color-coded sonography*.

**Figure 4:**
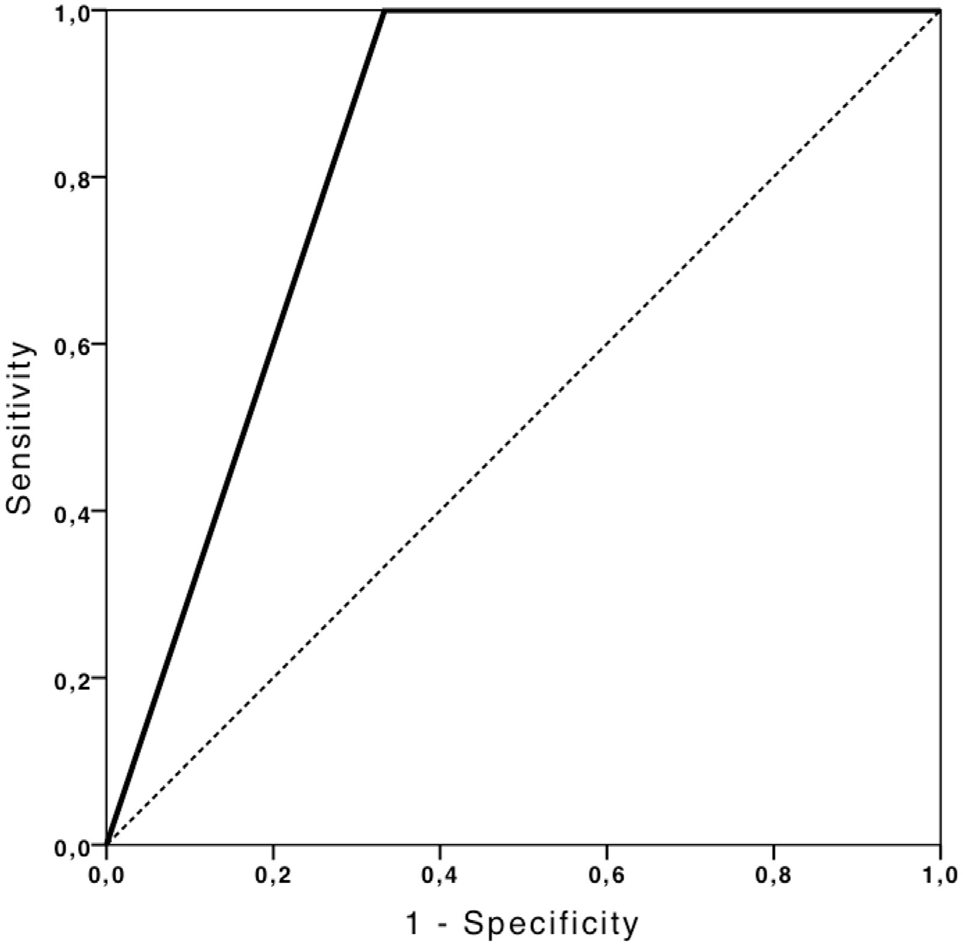
**ROC curve depicting the ability of TCCS to predict non-compressed peri-mesencephalic cisterns**.

**Figure 5:**
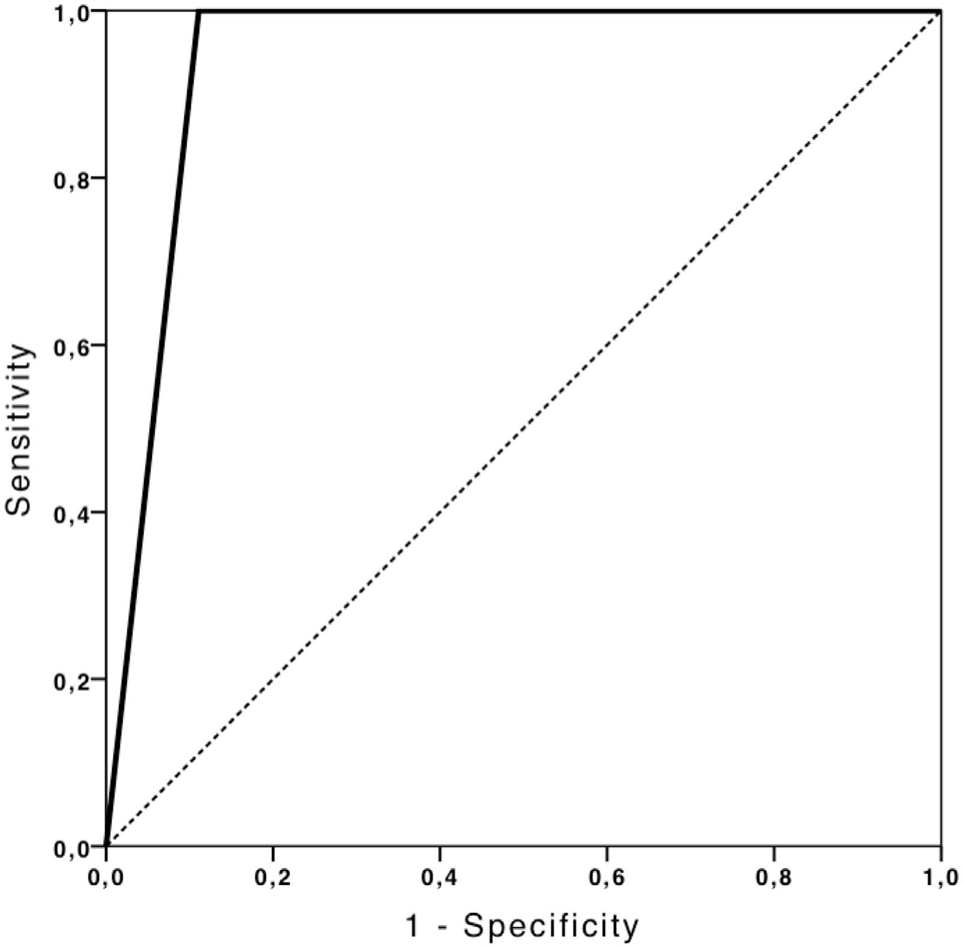
**ROC curve depicting the ability of TCCS to predict the presence of Sylvian fissure**.

TCCS showed a good performance in predicting non-compressed peri-mesencephalic cisterns (AUC: 0.83, 95% CI 0.46–1.0), with 100% sensitivity and 50% specificity (Figure 4). TCCS also presented the same performance in predicting the presence of the Sylvian fissure (AUC: 0.91, 95% CI 0.73–1.0), with 83% sensitivity and 100% specificity (Figure 5).

## Discussion

In this study, TCCS showed an excellent performance in predicting non-compressed peri-mesencephalic cisterns and the presence of the Sylvian fissure compared to brain CT scanning. We also observed a good correlation and agreement between TCCS and CT scanning regarding the shift of midline structures and the third ventricle width.

Recently, TCCS has been used increasingly frequently in the ICU environment because of its non-invasive characteristic, and it has become a reliable tool for assessing the brain parenchyma and cerebral vasculature in patients with an adequate acoustic window (13). Some authors have demonstrated that midline shifts could be reliably evaluated non-invasively at bedside in patients with intact skulls and with decompressive craniectomy (7). It has brought an opportunity to develop new forms of imaging for neuromonitoring without the risks of serial CT scans, especially in the transport of patients to the CT room.

In this study, we evaluated the TCCS ability to measure and predict some important findings that are usually seen only on brain CT scans. We observed an excellent correlation between the methods in measuring midline structure shifts, as demonstrated previously (11), and an excellent correlation in measuring the third ventricle width. This is an important finding and suggests some evidence about the cerebral complacency status because its absence could be interpreted as a high elastance cerebral system.

Another relevant and outstanding finding is the good performance in predicting non-compressed peri-mesencephalic cisterns, with high sensitivity (100%) and median specificity (50%). Usually, the peri-mesencephalic cistern’s obliteration on brain CT scan is interpreted as a strong predictor of intracranial hypertension. Thus, TCCS could lead to obtaining valuable knowledge at the bedside regarding the intracranial pressure status. Analogously, the excellent performance of TCCS in predicting the presence of the Sylvian fissure promotes the same information regarding good cerebral complacency, with 100% specificity and 83% sensitivity. To our knowledge, this is the first study to show the ability of TCCS in predicting peri-mesencephalic cisterns and Sylvian fissure status in neurocritical care patients.

This study has several limitations. First, the small number of patients could compromise the interpretation of the results. Second, although we did not identify any inadequate acoustic windows in our sample, it has been reported in 5–18% of patients (14, 15), which could be a limitation of our findings’ reproducibility. Finally, cerebral blood flow velocity and pulsatility index, which could be used as potential intracranial hypertension surrogates, were not evaluated because they were missing on our electronic database.

Thus, our findings showed that TCCS affords valuable information at the bedside in the neurocritical care setting. Its good accuracy in evaluating parameters that are surrogates for intracranial hypertension provides the much-needed possibility to manage severe traumatic brain-injured patients without the risks associated with patient transport and radiation dosage with a serial CT scan approach. Although we believe that more data are necessary to validate our results, the harmless and non-invasive nature of TCCS provides the feasibility to add it to the imaging armamentarium for the management of severe TBI.

## Ethics Statement

The study was approved by the *Comissão de Ética para Análise de Projetos de Pesquisa – CAPPesq*.

## Author Contributions

All authors listed, have made substantial, direct and intellectual contribution to the work, and approved it for publication.

## Conflict of Interest Statement

The authors declare that the research was conducted in the absence of any commercial or financial relationships that could be construed as a potential conflict of interest.
